# The Happy Campaign: Assessing the Effects of a Community-Wide Intervention on Older Adults Living in Public Housing

**DOI:** 10.1093/geroni/igaa057.1086

**Published:** 2020-12-16

**Authors:** Amy Gourley, Mignon Montpetit, LaKeesha James-Smith

**Affiliations:** 1 Illinois Wesleyan University, Carlock, Illinois, United States; 2 Illinois Wesleyan University, Bloomington, Illinois, United States; 3 Bloomington Housing Authority, Bloomington, Illinois, United States

## Abstract

In the field of developmental psychology, the Stress-and-Coping model (Lazarus & Folkman, 1984) posits that individual differences in biological, psychological, and social risk and protective factors serve to increase or buffer the impact of stressful experiences on psychological well-being later in life. Importantly, research suggests that residents of public housing generally experience more risk factors than elders at large (Rabins, et al. 1996). The present study examines the impact of a programmatic intervention, The Happy Campaign, on individuals living in public housing in a small Midwestern city (aged 51-90 years; Mage= 63.3 years; SDage= 8.6 years). Goals of the Happy Campaign were to improve residents’ coping skills and increase perceived support. Results demonstrated significant improvement in key aspects of well-being post-intervention; these included significant increases in exercise (p = .04), self-reported health (p = .01), as well as decreases in negative affect (p= .008). Data also show a moderate increase in residents’ hope post-intervention (p = .06). Although future research is needed to account for confounding variables that arose in conducting research in this community setting, these data provide preliminary evidence that a broad-based, environmental intervention may offset the myriad risks faced by particularly vulnerable elders, and even augment well-being.

